# Exercise as an Intervention to Reduce Study-Related Fatigue among University Students: A Two-Arm Parallel Randomized Controlled Trial

**DOI:** 10.1371/journal.pone.0152137

**Published:** 2016-03-31

**Authors:** Juriena D. de Vries, Madelon L. M. van Hooff, Sabine A. E. Geurts, Michiel A. J. Kompier

**Affiliations:** Behavioural Science Institute, Radboud University, Nijmegen, The Netherlands; University of Alabama at Birmingham, UNITED STATES

## Abstract

**Background:**

Many university students experience high levels of study-related fatigue. This high prevalence, and the negative impact of fatigue on health and academic performance, call for prevention and reduction of these symptoms. The primary aim of the current study was to investigate to what extent an exercise intervention is effective in reducing three indicators of study-related fatigue (emotional exhaustion, overall fatigue, and need for recovery). Effects of exercise on secondary outcomes (sleep quality, self-efficacy, physical fitness, and cognitive functioning) were also investigated.

**Methods:**

Participants were students with high levels of study-related fatigue, currently not exercising or receiving other psychological or pharmacological treatments, and with no medical cause of fatigue. They were randomly assigned to either a six-week exercise intervention (low-intensity running three times a week, *n* = 49) or wait list (no intervention, *n* = 48). All participants were measured before the intervention (T0), and immediately after the intervention (T1). Exercisers were also investigated 4 weeks (T2) and 12 weeks (T3) after the intervention.

**Results:**

Participants in the exercise condition showed a larger decrease in two of the three indicators of study-related fatigue (i.e., overall fatigue and need for recovery) as compared to controls. Additionally, sleep quality and some indicators of cognitive functioning improved more among exercisers than among controls. No effects were found for self-efficacy, and physical fitness. The initial effects of the exercise intervention lasted at follow-up (T2 and T3). At 12-week follow up (T3), 80% of participants in the exercise condition still engaged in regular exercise, and further enhancements were seen for emotional exhaustion, overall fatigue, and sleep quality.

**Conclusions:**

These results underline the value of low-intensity exercise for university students with high levels of study-related fatigue. The follow-up effects that were found in this study imply that the intervention has the potential to promote regular exercise and accompanying beneficial effects in the longer run.

**Trial Registration:**

Netherlands Trial Register NTR4412

## Introduction

University students are often faced with study stress, resulting from high study demands, and concern about academic grades [1,2]. When this study stress is prolonged and exceeds student’s adaptive resources, it can result in high levels of study-related fatigue [3–5] or in burnout (a more severe expression of study-related fatigue; [2]). A substantial number of university students experience study-related fatigue (e.g., estimated at 10% in the Netherlands—the country under study; [6]), and it is expected that this number will further increase [7], for instance due to increased performance demands (e.g., in the Netherlands, a minimum number of European credits is required in order to continue the study), and increased financial study costs (e.g. from 2015, in the Netherlands students no longer receive a governmental study gift, but a study loan, see: [8]). The prevalence of study-related fatigue, and its negative impact on health [9] and on academic performance [10], call for prevention and reduction of these complaints.

Evidence is emerging that regular exercise may be an accessible and inexpensive way to prevent or reduce (study-related) fatigue [11–14]. Both psychological and physiological working mechanisms may underlie potential positive effects of exercise on study-related fatigue. With respect to the former (see [15] for an overview), exercise may, for instance, help students to distract from (negative) thoughts about study demands (‘psychological detachment’; [16]). Detachment by means of exercise may enable students to return to a relaxed psychophysiological state that enhances the feeling of being refreshed by the start of a new (study) day [17–19]. Regarding the latter it is, for instance, hypothesized that individuals who exercise regularly—compared to those who do not exercise—show faster physiological (e.g., blood pressure) recovery from a stressor once the stressor is no longer present [20,21], which decreases the likelihood that persistent fatigue occurs [18]. The few available intervention studies concerning (study-related) fatigue show favorable effects of exercise, but research in this area can be advanced, as these studies did not include a control group [22] or focused on general fatigue instead of study-related fatigue [23]. To our knowledge, well designed randomized controlled trials to examine the efficacy of exercise for reducing study-related fatigue have as yet not been conducted. Therefore, the first aim of the current study was to investigate to what extent an exercise intervention (i.e., low intensity running, three times a week) is effective in reducing study-related fatigue. To this purpose, we carefully selected students with high levels of study-related fatigue and randomly assigned them to either an exercise intervention or wait list in order to establish to what extent the exercise intervention reduced fatigue (primary outcome) as compared to the natural course of time. In accordance with the proposed working mechanisms, and with available research pointing to the beneficial effects of exercise on fatigue [22,23], we expect that:

Hypothesis 1: The exercise intervention reduces study-related fatigue

Additionally, we aimed to obtain insight in the extent to which the exercise intervention is effective in improving four (secondary) outcomes relevant for students with high levels of study-related fatigue, to get a better understanding of a possible broader impact of the intervention. First, it was investigated whether exercise benefits sleep. Research shows that high levels of fatigue are linked to lower sleep quality [24]. For instance, it has been found that fatigued individuals spend less time in the sleep stages of slow wave and rapid eye movements sleep [25]. Spending enough time in these sleep stages is indicative of good sleep quality, and a requisite for feeling rested during the day and for adequate (academic) performance [26]. It has been argued that exercise promotes more sleep in these stages [27], although it is not exactly clear why this is the case. Several hypotheses have been proposed. One hypothesis is that the raised body temperature resulting from exercise is the link to better sleep quality (see for an overview of hypotheses: [27]). Based on proposed mechanisms and available evidence, we hypothesize:

Hypothesis 2a: The exercise intervention improves sleep quality

It has also been argued that exercise extends sleep duration, because the physical tiredness resulting from the effort expended during exercise may promote falling asleep [27]. Therefore, we expect:

Hypothesis 2b: The exercise intervention improves sleep duration

Second, it was investigated whether exercise benefits self-efficacy. Research shows that higher levels of fatigue are associated with lower self-efficacy [28]. It has been proposed that exercise generates self-efficacy through mastery experiences: individuals who are successful in fulfilling challenging tasks (such as exercise) by means of their own efforts develop abilities that foster confidence in themselves [29,30]. As the sample under study consists of students who did not engage in regular exercise before the intervention, we assume that exercise is a challenging task. Therefore, we expect that:

Hypothesis 2c. The exercise intervention improves self-efficacy

Third, it was examined whether the exercise intervention improves physical fitness. There is irrefutable evidence that regular exercise benefits physical fitness [31]. As we selected students who did not engage in regular exercise, we expect that gains can be obtained with respect to their physical fitness when they adopt a regular exercise pattern. Therefore, we propose:

Hypothesis 2d. The exercise intervention improves physical fitness

Fourth, the effect of exercise on cognitive functioning was studied. Fatigued individuals often report difficulties in everyday cognitive performance, such as slow thinking, and they perform worse on objectively measured cognitive functioning [32]. An emerging body of research points towards the idea that exercise improves (certain aspects) of cognitive functioning, especially the executive functions [33]. It has been argued that these improvements occur, since exercise stimulates the growth of new neurons (i.e. ‘neurogenesis’) in certain areas in the brain, such as the hippocampus that is associated with learning and memory [34]. Based on current insights, we expect:

Hypothesis 2e. The exercise intervention improves cognitive functioning

The final objective of this study was to investigate whether the effects of the exercise intervention would last on the longer term, and whether the follow-up effects were strongest for participants who spend more time on exercise during the follow-up period. To this purpose, we investigated whether intervention effects persisted at 4 weeks and 12 weeks after the intervention. Since we expect participants to have become accustomed to exercising regularly (i.e. to have developed an exercise-habit) and therefore continue running, we expect that:

Hypothesis 3a: The positive effects of the intervention are maintained at 4-week and 12-week follow-up.

As can be assumed that participants who exercise (more) regularly after the intervention are more exposed to the beneficial working mechanisms of exercise, we expect:

Hypothesis 3b: The follow-up effects are strongest for participants who spend more time on exercise during the follow-up period

## Methods

Participants were randomly assigned to one of two parallel groups, in a 1:1 ratio. It was investigated whether the exercise intervention was superior to wait list. The study protocol was approved by Ethical Commission Social Sciences of the Radboud University (registration number: ECSW2013-1811-142, see S1 and S2 Protocols). Additionally, the study protocol was registered in the Netherlands Trial Register before recruiting participants (NTR; see http://www.trialregister.nl): NTR4412. In the study protocol it was stated that participation in daily life (social interaction with family, friends, and student networks) would be assessed as well (i.e., secondary outcome). As we did not find a proper measure for this outcome, we preferred to not include this outcome in the current study.

### Eligibility criteria

Eligible participants were university students reporting high levels of study-related fatigue, defined by a score above validated cut-off points on two measures of (study-related) fatigue: ≥2.2 on the Emotional Exhaustion Scale of the Utrecht Burnout Scale for Students (UBOS-S; [2, 35]) and ≥ 22 on the Fatigue Assessment Scale (FAS; [36]). Participants were excluded if at the time of the study screening they a) exercised more than one hour a week; b) received psychological or pharmacological treatment for their fatigue complaints; c) reported a medical cause of their fatigue; d) were addicted to drugs, and e) were physically unable to run.

### Procedure

The study took place at the Radboud University (The Netherlands) from January 2014 to August 2014. Students were approached through different channels: by short recruitment talks during lectures, the Radboud University’s Research Participation System, social media, and flyers. Those interested in participation could fill out the UBOS (emotional exhaustion) and the FAS (overall fatigue) questionnaires on the study’s website (www.runtervention.nl). If participants scored ≥2.2 on the UBOS, and ≥22 on the FAS, they were asked to answer questions to assess the other criteria for eligibility. If they were eligible to participate, they visited the first author or a research assistant to read and sign informed consent, and to complete other baseline measures. Next, the randomization procedure was conducted.

### Randomization

The randomization procedure was conducted in a blocked fashion. We planned to deliver the intervention in groups of 10 participants. Of every block of 20 participants, 10 were allocated to the exercise intervention and 10 to the wait list. Participants were given a sealed and opaque envelope with the allocation. After opening the envelope, participants were allowed to tell the researcher in which group they were allocated.

### Exercise intervention

The exercise intervention comprised six weeks in which participants ran three times a week: twice a week in a group of ten people under supervision of a licensed running trainer and once independently (they were allowed to run with others). The running sessions took place outdoors on Tuesdays and Thursdays from 6 PM until 7 PM. Participants were instructed to run on a low intensity, in a pace that allowed them to have a conversation during running. The ability to converse during exercise has been shown to match low intensity exercise [37]. Low intensity was chosen because this intensity is preferable for lowering fatigue [23,38], and because it reduces the risk of injuries [39]. In addition, participants were advised to keep at least one day of rest (i.e. no running) in between the running sessions to diminish the risk of injuries. Furthermore, participants were told that the focus during running was not on running as long or as fast as possible, but rather on ‘feeling good’.

Two trainers supervised each running group, with each trainer supervising one running session a week. Trainers were all members of the Dutch Foundation of Running Therapy, a foundation focused on offering running to people with psychological complaints (www.runningtherapie-nederland.nl). Trainers had at least two years of experience in giving Running Therapy, and had been trained in psychopathology, physiology, and running training principles. Trainers were instructed to observe participants’ running intensity (i.e. make sure that all participant were able to have a conversation during running) and keep the focus on ‘feeling good’.

Each running session comprised 60 minutes: a warming up of about 15 minutes (running on a low intensity alternated with walking and flexibility exercises), a core program consisting of running alternated with walking of about 30 minutes, and a cooling down of about 15 minutes. During the six weeks, the periods of running in each running session were extended, and the walking periods shortened, so that after six weeks the participants were able to run 20 minutes uninterrupted on a low intensity.

### Control condition

During the six weeks of the exercise intervention, the participants in the control condition were on a wait list and thus received no intervention. After these six weeks, they were given the opportunity to follow the exercise intervention as well.

### Primary and secondary outcomes

Primary and secondary outcomes were measured pre (T0), and post (T1) intervention among all participants. Those in the experimental condition were also measured at follow-up: 4 weeks (T2), and 12 weeks after the intervention period (T3). At follow-up, physical fitness was only measured at T3, since 4 weeks constitutes a relatively short time to notice differences in physical fitness [40]. Due to possible learning effects [41], cognitive functioning was not measured at follow-up.

#### Study-related fatigue

Study-related fatigue was measured with three indicators: emotional exhaustion, overall fatigue and need for recovery. We based the inclusion criteria on the first two indicators only, since a validated cut off score for need for recovery does not exist.

Emotional exhaustion was measured with a Dutch adaptation of the Maslach Burnout Inventory (MBI; [42]): Utrecht Burnout Scale [35]. We used a modified version that was especially developed for students (UBOS-S; [2]). From this questionnaire, the ‘Exhaustion’ scale was used, consisting of 5 items, answered on a 7-point Likert scale. An example question is: “I feel burned out from my studies” (0 = *never*, 6 = *always; every day*). A mean score was computed. A mean score ≥ 2.2 is considered as ‘high’ emotional exhaustion [35]. Cronbach’s alpha ranged between .81 (T1) and .90 (T4).

Overall fatigue was measured with the 10-item Fatigue Assessment Scale (FAS), developed and validated by Michielsen, de Vries & Van Heck [36]. An example question is: “I am bothered by fatigue”. Items were rated on a 5-point Likert-scale (1 = *never*, 5 = *always*). A sum score was computed, and a score of ≥ 22 is determined as a ‘high’ level of fatigue [38]. Cronbach’s alphas ranged between .79 (T1) and .88 (T4).

Need for recovery was assessed with the 6-item ‘Need for Recovery Scale’ [43]. We adapted the scale for students, meaning that ‘work’ was replaced by ‘study’. An example item is “I find it hard to relax at the end of a day of studying”, and all items were rated on a 4-point Likert scale (1 = *(almost) never*, 4 = *(almost) always*). A mean score was computed. Cronbach’s alpha ranged from .75 (T1) and .87 (T4).

#### Sleep

To assess sleep quality, a sum score was computed of six items adapted from the sleep quality scale of the Questionnaire on the Experience and Evaluation of work (VBBA; [44]). The questions tapped into the three main components of insomnia [45]: ‘difficulty initiating sleep’ (1 item, i.e., “I have difficulties falling asleep”), ‘difficulty maintaining sleep’ (2 items, e.g. “I often wake up during the night”), and ‘non-restorative sleep’ (2 items, e.g. “Most of the time, I feel refreshed when I wake up” [reversed]). Furthermore, there was one overall question for sleep quality: “I often sleep well”. All items had a dichotomous answer category: 1 = *yes*, 0 = *no*. Positively formulated items were reversed, and a sum score was calculated, so that a higher score indicates more sleep complaints. Cronbach’s alphas ranged between .61 (T1) to .76 (T4). In addition, sleep quantity (mean hours of sleep a night) was questioned.

#### Self-efficacy

To measure self efficacy, the 12-item General Self Efficacy Scale was used (GSES-12; [46]). An example item is: ‘When I make plans, I am certain I can make them work’. A mean score was computed of all items, rated on a 5-point Likert scale (1 = *strongly disagree*, 5 = *strongly agree*). Cronbach’s alphas ranged between 0.80 (T1) and 0.86 (T2).

#### Physical fitness

Physical fitness was measured with the Conconi test [47]. Although the test received some critics (see [48]), it has been argued that it provides an appropriate indicator of the heart rate deflection point on which physical fitness can be assessed [49]. The test was performed on a stationary cycle ergometer in the Radboud University Sport Centre. Participants wore a heartrate belt, and were instructed to cycle as long as physically possible and to keep their cadence between 70–80 rpm. Before starting the actual test, participants completed a warm-up procedure, consisting of 4 minutes of low-intensity cycling. The start intensity (power—the number of watts) was based on weight and age and thus differed between participants. During the test, the wattage gradually increased, and the time cycling per watt interval gradually decreased. After finishing the test, the heart rate deflection point was determined by the software (e.g. the point at which the heart rate—power relationship deviates from linearity; see [48]). Estimated Vo_2_max ml/kg/min was based on the heart rate deflection point, the number of watts, age, and gender [47]. Completion of the test took about 20 minutes.

#### Cognitive functioning

Cognitive functioning was measured with one self-report measure, and three objective performance tests. These tests measured three types of executive functioning: updating, inhibition, and switching [50].

Self-reported cognitive functioning was measured with the Dutch translation of the Cognitive Failures Questionnaire (CFQ; [51]). It consists of 25 items rated on a 5-point Likert scale (1 = *never*, 5 = *very often*), and an example question is: “Do you find you forget appointments?”. A mean score was computed. The reliability of the scale ranged between α = 0.83 (T1) and α = 0.90 (T2).

Updating refers to constant monitoring and fast addition/deletion of information in working-memory [52] and was assessed by the 2-Back task [53]. The test consisted of 284 letters that were displayed one-by-one in the centre of a computer screen. The letters were ‘b’, ‘d’, ‘g’, ‘p’, ‘t’, and ‘v’ and were both displayed as capital and small letters. Stimulus duration was set at 450 ms with the interval between two stimuli fixed on 750 ms. Once a letter appeared that was similar to a letter that had appeared two stimuli before, participants had to push a button on a button-box (target rate 32.5%). For a correct response, no distinction was made between capital and small letters. Completion of the test took about seven minutes. The number of correct responses was used as a measure for updating.

Inhibition addresses the suppression of dominant but irrelevant automatic responses to stressors [52] and was measured by the Sustained Attention to Response Test (SART; [54]). A total of 450 digits (ranging from 1–9) were displayed one-by-one in the centre of a computer screen in a random fashion. Participants had to push a button on a button-box each time when they saw a digit, except if the digit was ‘3’ (target rate 11.1%). Digits were displayed for 250 ms and the interval between digits was fixed at 850ms. Completion of the test took about eight minutes. The number of ‘correct inhibitions’ (not pressing the button when a ‘3’ was displayed) constituted the measure for inhibition.

Switching refers to the ability of shifting between different tasks [52] and was measured with the Matching task [55]. In each trial of the test, four different geometric figures (circle, hexagon, square and triangle) were displayed in four different colours (blue, green, red and yellow) in the lower half of the screen. Also, a coloured reference geometric figure was shown in the upper half of the screen. The participants had to match the reference figure (in the upper half of the screen) to one of the four figures (in the lower half of the screen) according to shape or colour. The combination between colour and shape figures was presented in such a way that there was one correct answer. Whether participants had to match according to shape or colour, was (randomly) indicated by a cue that was displayed for 1000ms. Participants could push one of the four buttons on a keyboard that corresponded to each of the four match figures in the lower half of the screen. Participants had to push the button as fast as possible. The whole test consisted of 31 task runs, each consisting of on average six trials (range: 4–8 trials). For all the trials during one task run, one cue was given (matching according to shape or colour). During the test, half of all task runs consisted of ‘switch’ runs, in which the type of cue differed from the previous run. The other half consisted of ‘repetition’ runs, in which the type of cue was identical to the previous run. The duration of the test was about six minutes. The reaction time of the first trial of the ‘switch’ or the ‘repetition’ runs was used as a measure for ‘switching’ and ‘repetition’ respectively. Runs in which the cue was not correctly followed or in which no response was given, were not included in the analyses.

To obtain a full assessment of cognitive functioning, we additionally explored the subjective costs (fatigue, motivation, demands, and effort) associated with performing the cognitive tests [56–58]. Before doing the cognitive tests, participants rated how motivated they were to do the tests. Fatigue was measured prior to and after the tests. After completing all the tests, participants were asked how demanding the tests had been, and participants indicated how much effort they spent when doing the tests. The subjective costs of doing the cognitive tests were measured by using single item measures, answered on a scale from 1 (*not at all*) to 10 (*very much*).

#### Exercise behaviour

At T4 (follow-up after 12 weeks), participants in the exercise condition were asked to indicate how frequently and how many minutes a week they exercised in general during the 12-week follow-up period after the intervention.

### Power analysis

To determine the number of participants, a power analysis was conducted in the program G*Power [59]. This analysis was based on a repeated measures MANOVA (RM-MANOVA) with time as within subjects factors and condition as between subjects factor. This analysis showed that a total of 90 participants was required in order to detect a medium effect of .30 (Pillai’s V) on study-related fatigue outcome from pre to the immediate post intervention, given a two-sided 5% significance level and a power of 80%. A comparable previous study showed a medium effect size as well [23]. Because we anticipated a dropout rate of about 20%, we intended to recruit 108 participants. During the study, however, the dropout turned out to be low. Consequently, it was decided to stop recruiting after the number of 100 participants had been reached (five blocks of 20 participants).

### Statistical analyses

We used SPSS version 19 to analyze the data [60]. The statistical analyses were based on the intention-to-treat principle [61]. This means that all participants who were randomized were included in the analyses (i.e., also those who ended their participation before or during the intervention period).

Pearson correlations (*r*) were used to explore whether the three indicators of study-related fatigue were inter-related. In order to test whether the exercise intervention was effective in reducing these three indicators of study-related fatigue (*H1*), a RM-MANOVA was conducted with ‘time’ (pre [T0] versus post intervention [T1]) as within-subjects factor and ‘condition’ as between-subjects factor (‘intervention’ versus ‘wait list’). For the effects of the exercise intervention, we were interested in the Group × Time interactions of these RM-(M)ANOVAs, since an interaction indicates that the change in the outcome over time is different between conditions.

Additionally, to investigate the extent to which the intervention resulted in clinical meaningful changes [62] in the primary outcomes emotional exhaustion and fatigue, a Chi Square Test was performed to examine if the proportion of participants that were ‘recovered’ at T1 (for burnout: <2.2 on the UBOS; for fatigue: <22 on the FAS) differed between the exercise and the wait list condition. An effect size (φ) between .2–.5 was considered as small, .5–.8 as medium, and >.8 as large [63].

Separate repeated measures (M)ANOVAs were done with ‘time’ (pre [T0] versus post intervention [T1]) as within-subjects factor and ‘condition’ as between-subjects factor (‘intervention’ versus ‘wait list’) to investigate the effect of the exercise intervention on sleep (*H2a* and *H2b*), self-efficacy (*H2c*), and physical fitness (*H2d*). The four indicators of cognitive functioning (*H2e*), and subjective costs were also separately analyzed by means of a (M)ANOVA. The Matching Task was analyzed using a 2 × 2 × 2 mixed design with ‘run type’ (switch versus repetition) and ‘time’ (pre [T0] versus post intervention [T1]) as within factors and ‘condition’ as between factor (‘intervention’ versus ‘wait list’).

To find out whether intervention-effects would persist during the follow-up period (*H3a*) among participants in the exercise condition (i.e. participants in the control condition were not measured at follow up, because during this period they received the exercise intervention), separate RM-(M)ANOVAs for the outcomes were performed with ‘time’ (pre [T0] vs. post intervention [T1] vs. 4 weeks after the intervention period [T2] vs. and 12 weeks after the intervention period [T3]) as within-subjects factor. Since physical fitness was not measured at T2, the RM-ANOVA for this outcome included three time points (T0, T1, and T3). If the time effect of the RM-(M)ANOVA was significant, post hoc tests were conducted to exactly determine between what time points the outcome had changed. Since Bonferroni correction for multiple comparisons is often too conservative, we used the Sidak correction instead [64].

For all RM- (M)ANOVAs, an effect size (η^2^) between .01–.06 was considered as small, .06–.14 as medium, and > .14 as large [65]. Significant interactions effects were further examined by means of paired t-tests, for which Cohen’s *d* was used as an effect size.

In order to investigate whether follow-up effects were moderated by the amount of exercise participants engaged in during the follow-up period (*H3b*), the amount of exercise during this 12 week follow up period (number of minutes a week) was added as covariate in a RM-(M)ANCOVA with ‘time’ (post intervention [T1] versus follow up after 12 weeks [T3]) as within factor. We were interested in the interaction between the amount of exercise and time, since this indicates that the development over time in a certain outcome is moderated by the amount of exercise participants engage in. The follow up effects at 4 weeks after the intervention (T2) were not taken into account, because exercise was measured as the ‘mean number of minutes a week during the 12 weeks after the intervention’.

## Results

### Descriptives

The baseline characteristics of participants in each of both conditions are in Table 1. There were no differences between conditions at baseline. The participants were recruited in the period of February 2014 to May 2014.

**Table 1:** Background characteristics at study entry.

	Exercise condition(*n* = 50)	Control condition(*n* = 49)
Age, years (mean ± *SD*)	20.9 ± 2.5	20.7 ± 2.2
Male *n* (%)	9.0 (18.0)	10.0 (20.4)
Dutch *n* (%)	39.0 (78.0)	33.0 (67.3)
German *n* (%)	11.0 (22.0)	16.0 (32.7)
Hours of study a week (mean ± *SD*)	26.9 ± 12.5	26.7 ± 10.8
Study: Psychology *n* (%)	37.0 (74.0)	34.0 (69.4)
Study enjoyment (mean ± *SD*)^a^	7.8 ± 0.8	7.9 ± 1.1
Additional job *n* (%, mean hours ± *SD*)	22.0 (44.0, 6.6 ± 3.9)	21.0 (42.9, 9.1 ± 5.3)

^a^ 1 = no enjoyment at all, 10 = very high level of enjoyment

**Fig 1:**
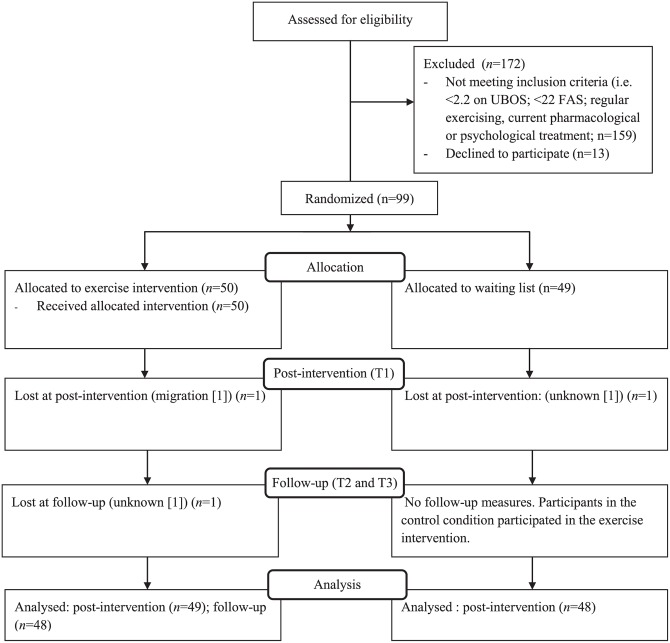
Participant flow diagram.

The participant flow diagram is depicted in Fig 1. For the self-report measures, 49 of 50 participants in the exercise condition and 48 of 49 participants in the control condition completed all questionnaires. However, not every participant completed the physical fitness test. Reasons were: injury (*n* = 3 [controls]), not willing to do the test for unknown reasons (*n* = 3), not able to do the exercise due to extreme fatigue (*n* = 1), and not enough data points to estimate the Vo_2_max due to very low physical fitness (*n* = 1).

In the exercise condition, four participants dropped out during the intervention: reasons were injuries (*n* = 3) and migration (*n* = 1). Those who got injured, continued to participate in the measurements of the study. Overall, the compliance during the exercise intervention was high: on average 14.16 (*Standard Deviation [SD]* = 3.61) of the in total 18 running sessions were carried out.

In the control condition, most participants did not exercise during the intervention period. Only two participants in the control condition started to engage in regular exercise, for on average 75 minutes and 100 minutes a week respectively. After the intervention period, 40 of the initial 49 participants in the control condition participated in the exercise intervention.

### Effects on primary outcome (Hypothesis 1)

#### Study-related fatigue

The three indicators of study-related fatigue were significantly inter-related (at T0: emotional exhaustion and overall fatigue *r* = .63; emotional exhaustion and need for recovery *r* = .35; overall fatigue and need for recovery *r* = .36). A significant multivariate Group × Time interaction effect was found for the combination of the three indicators of study-related fatigue (F(3,93) = 3.00, *p* = .026, *η*^2^ = .10). As the multivariate test was significant, we examined univariate main effects. In Table 2, the Group × Time interaction for emotional exhaustion was not significant, but Group × Time interactions reached significance for overall fatigue (*F* = 4.85, η^2^ = .05) and need for recovery (*F* = 7.66, η^2^ = .08). Participants in the exercise condition showed a stronger decrease in overall fatigue (*t*(48) = 6.82, *p* = < .001; Cohen’s *d*: 0.90) than those in the control condition (*t*(47) = 3.08, *p* = .003; Cohen’s *d*: 0.46). Additionally, the exercise intervention resulted in a stronger decrease over time in need for recovery (*t*(48) = 7.42, p <.001; Cohen’s *d*: 0.84) compared to the control condition (*t*(47) = 2.59, p = .013; Cohen’s *d*: 0.42).

**Table 2:** Mean (SD) scores of emotional exhaustion, overall fatigue, need for recovery, sleep quality, sleep duration, self-efficacy, and physical fitness pre (T0) and post intervention (T1), at follow-up after 4 weeks (T2) and at follow-up after 12 weeks (T3) per condition.

Outcome(theoretical range)	T0		T1		Intervention effects			T2	T3	Follow-up effects		
	Exercise*M (SD)*	Control *M (SD)*	Exercise *M (SD)*	Control *M (SD)*	Effect	*F*^a^	η^2^	Exercise *M (SD)*	Exercise *M (SD)*	*F*^b^	Effect^c^	Δ*M*
Emotional Exhaustion (0–6)	3.74 (1.05)	3.78 (0.84)	2.76 (1.16)	3.03 (1.06)	G	0.75	.01	2.57 (1.03)	2.27 (1.09)	38.65**	**T0 vs T2**	1.16**
					T	66.15**	.41				T1 vs T2	0.24
					G×T	1.23	.01				**T0 vs T3**	1.46**
											**T1 vs T3**	0.53*
											T2 vs T3	0.29
Overall fatigue(10–50)	30.47 (5.52)	30.92 (4.91)	25.35 (5.86)	28.29 (6.52)	G	2.78	.03	25.46 (5.64)	23.31 (5.50)	38.51**	**T0 vs T2**	4.89**
					T	46.67**	.33				T1 vs T2	0.06
					**G×T**	4.85*	.**05**				**T0 vs T3**	6.92**
											**T1 vs T3**	2.09*
											**T2 vs T3**	2.02*
Need for Recovery (1–4)	2.55 (0.61)	2.36 (0.50)	2.05 (0.58)	2.15 (0.49)	G	0.19	.00	2.06 (0.59)	1.97 (0.61)	26.56**	**T0 vs T2**	0.49**
					T	45.53**	.32				T1 vs T2	0.00
					**G×T**	7.66*	.**08**				**T0 vs T3**	0.57**
											T1 vs T3	0.09
											T2 vs T3	0.09
Sleep quality (0–6)	3.08 (1.62)	2.71 (1.40)	2.16 (1.64)	2.75 (1.64)	G	0.15	.00	2.11 (1.90)	1.70 (1.79)	15.86**	**T0 vs T2**	-0.94**
					T	7.85*	.08				T1 vs T2	-0.11
					**G×T**	9.41*	.**09**				**T0 vs T3**	−1.34**
											**T1 vs T3**	−0.51*
											T2 vs T3	-0.40
Sleep duration (hours)	7.39 (1.10)	7.50 (1.80)	7.45 (0.87)	7.42 (0.90)	G	0.05	.00	7.43 (0.77)	7.47 (1.04)	0.16	T0 vs T2	0.06
					T	0.02	.00				T1 vs T2	-0.02
					G×T	0.38	.01				T0 vs T3	0.08
											T1 vs T3	0.04
											T2 vs T3	0.04
Self efficacy (1–5)	3.31 (0.52)	3.23 (0.58)	3.38 (0.59)	3.17 (0.61)	G	1.75	.02	3.41 (0.51)	3.44 (0.54)	1.49	T0 vs T2	-0.08
					T	0.04	.00				T1 vs T2	-0.04
					G×T	2.92	.03				T0 vs T3	-0.10
											T1 vs T3	-0.05
											T2 vs T3	-0.02
Vo_2_max^d^	28.45 (5.46)	27.93 (4.14)	29.39 (5.19)	28.35 (3.95)	G	0.67	.01	^e^	29.65 (5.40)	2.67	T0 vs T3	-1.11
					T	4.69*	.05				T1 vs T3	-0.26
					G×T	0.63	.01					

*Note*. Relevant effects are in bold; *M* = Mean, *SD* = Standard Deviation, *F* = F-statistic, *ΔM* = change in mean, η^2^ = effect size, G = Group effect, T = Time effect; G×T = Group × Time effect

^a^ Univariate effect of (M)ANOVAs with ‘time’ (pre vs. post) as within subjects factor and ‘condition’ (exercise vs. control) as between subjects factor

^b^ Time effect pre (T0) vs post (T1) vs 4 weeks after the intervention (T2) vs 12 weeks after the intervention (T3), only for the exercise condition

^c^Post hoc tests using Sidak correction

^d^ 8 missing values

^e^ Vo_2_max was not assessed at T2

* *p* = <.05

** *p* = <.01

Table 3 shows the extent to which the intervention resulted in clinical meaningful changes in emotional exhaustion and overall fatigue (i.e. participants who recovered from T0 to T1; for emotional exhaustion: <2.2 on the UBOS; for fatigue: <22 on the FAS). Chi Square tests revealed that, for emotional exhaustion, the proportion of recovered participants was marginally higher in the exercise condition than in the control condition (χ^2^(1) = 3.72, *p* = .054, φ = .196). For overall fatigue, the proportion of recovered participants was higher in the exercise condition (χ^2^(1) = 4.738, *p* = .036, φ = .212). Taken together, the results generally support *Hypothesis 1*, as we found that participants in the exercise intervention showed a larger decrease in overall fatigue and need for recovery over time compared to the wait list controls.

**Table 3:** Stability and change: percentages of participants who improved, recovered, unimproved or deteriorated on emotional exhaustion, and overall fatigue from T0 to T1.

	Improved *n* (%)	Recovered *n* (%)	Unimproved or deteriorated *n* (%)
Exercise condition (*n* = 49)			
Emotional exhaustion	42 (85.7)	19^1^ (38.8)	7 (14.3)
Fatigue	40 (86.0)	16^2^ (32.7)	9 (18.4)
Control condition (*n* = 48)			
Emotional exhaustion	37 (77.1)	10^1^ (20.4)	11 (22.9)
Fatigue	33 (68.8)	7^2^ (14.6)	15 (31.3)

Note.

^1^ lower than 2.2 on the UBOS at T1

^2^ lower than 22 on the FAS at T1

### Effects on secondary outcomes (Hypotheses 2a to 2e)

#### Sleep

A significant multivariate Group × Time interaction effect was found for sleep (F(2,94) = 4.73, *p* = .011, *η*^2^ = .09). Univariate tests (see Table 2) revealed a significant Group × Time interaction for sleep quality (*F* = 9.41, η^2^ = .09), in support of *Hypothesis 2a*. T-tests showed that sleep quality improved in the exercise condition (*t*(48) = -3.97, p <.001, Cohen’s *d*: 0.56), but not in the control condition (*t*(48) = 0.20, p = .844, Cohen’s *d*: -0.03). In the absence of a significant Group × Time interaction effect for sleep duration, *Hypothesis 2b* was not supported.

#### Self-efficacy

For self-efficacy, no significant Group × Time effect was found (*p* = .091; see Table 2). Hence, *Hypothesis 2c* was not supported.

#### Physical fitness

No Group × Time effect for physical fitness was found (see Table 2). Therefore, *Hypothesis 2d* was not supported.

#### Cognitive functioning

Table 4 shows the results of the RM-(M)ANOVAs for cognitive functioning. A significant Group × Time interaction was found for self-reported cognitive functioning (*F* = 26.60, η^2^ = .22). T-tests revealed that ‘exercisers’ showed a decrease in cognitive failures over time (*t*(48) = 5.85, *p* = < .001, Cohen’s *d*: 0.87), while no such change was found in the control condition (*t*(47) = -0.80, *p* = .429, Cohen’s *d*: -0.07).

**Table 4:** Results for cognitive functioning pre (T0) and post intervention (T1) per condition.

Outcome (Theoretical range) [Task used]	Pre (T0)		Post (T1)				
	Exercise*M* (*SD*)	Control*M* (*SD*)	Exercise*M* (*SD*)	Control*M* (*SD*)	Effect	*F*	*η*^*2*^
Cognitive Failures (1–5)	2.90 (0.44)	2.75 (0.45)	2.51 (0.46)	2.79 (0.61)	G	.49	.01
					T	17.72**	.16
					**G×T**	26.60**	.**22**
Updating [2-Back Task]^a^	62.16 (12.20)	65.77 (12.81)	73.07^1^ (11.72)	74.42^2^ (13.12)	G	1.02	.01
					T	83.68**	.49
					G×T	1.12	.01
Inhibition [SART]^a^	21.17 (7.12)	24.69 (7.79)	24.37^3^ (8.42)	24.14^4^ (8.76)	G	1.44	.02
					T	1.03	.01
					**G×T**	5.98*	.**07**
Switching [Matching Task]							
*RT repetition*^b^	929.51 (183.36)	923.33 (164.83)	800.74 (129.54)	838.05 (129.54)	G	0.04	.00
					T	67.07**	.42
*RT switch* ^b^	1014.88 (187.21)	1013 (219.79)	908.87 (152.27)	902.68 (167.43)	Rt	61.35**	.40
					RtxG	0.74	.01
					RtxT	0.01	.00
					G×T	0.53	.01
					RtxG×T	1.38	.02
Motivation(1–10)	8.27 (1.51)	8.05 (1.40)	8.06 (1.74)	7.93 (1.45)	G	0.37	.00
					T	1.44	.01
					G×T	0.13	.01
Fatigue (1–10)							
*Before*^c^	6.35 (1.71)	5.73 (2.02)	4.77 (2.02)	5.73 (2.18)	G	0.24	.00
					T	10.54*	.11
					**G×T**	10.54*	.**08**
*After*^d^	7.31 (1.36)	7.07 (1.50)	6.23 (1.92)	7.11 (1.61)	G	1.17	.01
					T	5.58*	.07
					**G×T**	8.50*	.**08**
*Difference*^e^	0.96 (1.38)	1.27 (1.91)	1.46 (1.65)	1.39 (1.73)	G	0.18	.00
					T	2.47	.03
					G×T	1.71	.01
Demands (1–10)	7.47 (1.22)	7.35 (1.07)	6.73 (1.37)	7.20 (1.08)	G	0.64	.01
					T	16.17**	.16
					**G×T**	7.32*	.**08**
Effort (1–10)	7.87 (1.35)	7.68 (1.18)	7.79 (1.66)	7.59 (1.45)	G	0.52	.01
					T	0.53	.01
					G×T	0.00	.00

*Note*. *M* = Mean, *SD* = Standard Deviation, *F* = F-test, *ΔM* = change in mean, η^2^ = effect size, G = Group effect, T = Time effect; G×T = Group × Time effect

^a^ Correct responses

^b^ Reaction times in milliseconds

^c^ Score before making the cognitive tests

^d^ Score after making the cognitive tests

^e^ Difference score between fatigue before and after making the cognitive tests

^1^ 4 missing values

^2^ 5 missing values

^3^ 7 missing values

^4^ 11 missing values

* *p* = <.05

** *p* = <.01

Mixed results were found for the cognitive tests. It is important to note that technical problems caused inadequate recording of the T1-reaction times for some participants: for 9 participants with respect to the 2-back test (updating), and for 17 participants with respect to the SART (inhibition). Analyses were only conducted for participants with adequate recordings. For inhibition (SART) we found a significant Group × Time interaction (*F* = 5.98, η^2^ = .07). Additional t-tests showed that participants in the exercise condition improved (*t*(40) = -2.35, *p* = .024, Cohen’s *d*: 0.41), whereas those in the control condition did not (*t*(36) = 1.08, *p* = .290, Cohen’s *d*: -0.06). No significant Group × Time interactions for updating (2-back test) and switching (Matching Task) were found.

Table 4 also shows the subjective costs of cognitive test performance. The RM-MANOVA revealed a significant multivariate effect of the combined subjective costs (F(5,86) = 3.88, *p* = .003, *η*^2^ = .18). Considering univariate effects, both at T0 and T1, all participants were motivated to conduct the cognitive tests (mean scores ranging from 7.93 to 8.27), and participants’ motivation did not change (over time) between conditions (no significant ‘Group’ and ‘Group × Time’ interaction). There were significant interaction effects for ‘fatigue’ before (*F* = 10.54, η^2^ = .08) and after (*F* = 8.50, η^2^ = .08) the intervention. T-tests indicate that exercisers, over time, became less fatigued before performing the cognitive tests (*t*(47) = 4.35, *p* = <.001, Cohen’s *d*: 0.84) and also less fatigued after having performed the cognitive tests (*t*(47) = 3.70, *p* = .001, Cohen’s *d*: 0.65), when compared to controls (before: *t*(46) = 0.36, *p* = .719), Cohen’s *d*: 0, and after: *t*(45) = -0.15, *p* = .878, Cohen’s *d*: -0.02). The non-significant “difference” interaction effect indicates that both groups became equally tired from the test battery at both points in time. A significant Group × Time interaction was found for demands though (*F* = 7.32, η^2^ = .08). Exercisers considered the tests less demanding over time (*t*(47) = 4.48, *p* <.001), while controls did not (*t*(44) = 1.23, *p* = .227). The non-significant Group × Time interaction of ‘effort’ indicates no across time difference for the amount of effort expended when conducting the cognitive tests. Taking these results together, we found partial support for *Hypothesis 2e* (decreased cognitive failures, increased inhibition, and decreased demands during the tests).

### Follow-up effects (Hypothesis 3a)

Table 2 shows the means and standard deviations of the primary and secondary outcomes at 4 weeks (T2) and 12 weeks (T3) after the intervention, for the exercisers only (the controls could no longer serve as controls during the follow-up as they started to exercise themselves). For the three combined indicators of study-related fatigue, repeated measures MANOVA showed a large multivariate effect of ‘time’ (*F*(9,38) = 13.16, *p* <.001, *η*^2^ = .76). Univariate effects (see Table 2), were found for all three indicators (emotional exhaustion: *F* = 38.65, η^2^ = .46; overall fatigue: *F* = 38.51, η^2^ = .46; need for recovery: *F* = 26.56, η^2^ = .37). Post host tests revealed that exercisers showed a decrease in emotional exhaustion, overall fatigue and need for recovery from baseline (T0) to follow-up (both at T2 and T3). In addition, emotional exhaustion and overall fatigue further decreased from T1 (post intervention) to T3 (12 weeks after the intervention).

With respect to sleep, we found a large significant multivariate effect (*F*(6,41) = 5.37, *p* <.001, *η*^2^ = .44). As can be seen in Table 2, the univariate effect of sleep quality was significant (*F* = 15.86, η^2^ = .26). No change over time was found for sleep duration. Post hoc tests displayed an increase in sleep quality from baseline (T0) to follow-up (both at T2 and T3), and from post intervention (T1) to 12 weeks after the intervention (T3). No significant time effects were found for self-efficacy and physical fitness, meaning that these outcomes did not change over time during follow up among the exercisers.

In summary, we found further improvements from post intervention to 12 weeks after the intervention in emotional exhaustion, overall fatigue and sleep quality. Therefore, we conclude that *Hypothesis 3a* is partially supported.

### Exercise maintenance in relation to follow-up effects (Hypothesis 3b)

A total of 40 participants (from 50 in the exercise condition) engaged in regular exercise during the follow up period, for an average of 113.93 minutes (SD = 88.51) a week, in on average 2.55 (*SD* = 1.34) exercise sessions a week.

For study-related fatigue, sleep, and self-efficacy, we did not find significant interaction effects between the covariate ‘exercise’ and ‘time’ (*F*’s ranging from to 0.00 to 2.88, all *p*’s > .05). This means that follow-up effects were not moderated by the amount of exercise participants engaged in during the follow-up period. For physical fitness, the RM-ANCOVA showed a significant interaction between time and exercise *F*(1,43) = 4.53, *p* = .039). To interpret this interaction effect, we constructed two subgroups based on the minutes of exercise a week: < 60 minutes, and ≥ 60 minutes a week. Inspecting the means of these groups revealed that exercisers with ≥ 60 minutes of exercise during the follow-up period showed an increase in physical fitness compared to less active participants. Therefore, we conclude that *Hypothesis 3b* is partly supported, since we only found support for this hypothesis with respect to physical fitness.

## Discussion

The aim of the present study was to investigate: i) to what extent an exercise intervention was effective in reducing study-related fatigue (primary outcome) among university students; ii) whether the exercise intervention was able to improve four secondary outcomes that are related to high levels of study-related fatigue (sleep, self-efficacy, physical fitness, and cognitive functioning); and iii) whether the effects of the exercise intervention were maintained on the longer term.

As to the first aim, it can be concluded that the exercise intervention is effective in reducing study-related fatigue. We found that—compared to controls—‘exercisers’ showed a larger decrease in two of the three indicators of study-related fatigue (i.e., overall fatigue and need for recovery) after the intervention period. Additionally, we showed that exercisers fell more often below validated cut-off scores of overall fatigue after the intervention period as compared to controls, implying that the changes found in overall fatigue can be considered clinically meaningful [62]. By supporting a relation between exercise and reduced levels of fatigue, these findings extend previous research also showing an inverse relationship between these two constructs (e.g. [23,66]). It is probable that biological (e.g., faster cardiovascular recovery after stress exposure; [20]) as well as psychological mechanisms (i.e., psychological detachment; [16]) are responsible for this relation. Our study, however, offers no definitive conclusions about the exact mechanisms that may mediate the reduction in study-related fatigue among exercisers.

One unanticipated finding was that exercisers did not show a larger improvement in emotional exhaustion than the control group. Instead, both groups showed a decrease over time in this outcome. This decrease in both groups may be confounded by the timing of this study during the course of the study year. That is, the post-intervention measurements were done in the period between May and July, and in this period (in the Netherlands) students know whether they have earned enough European credit points to be admitted to the next study year. This may decrease study stress and accompanying fatigue. As—compared to the other two measures of fatigue—the emotional exhaustion-measure is most explicitly related to fatigue as a consequence of the study, this may contribute to the decrease in this outcome in both groups.

As to our second aim, the exercise intervention proved to be effective in improving sleep quality. Inspection of the separate items of the sleep quality scale revealed that especially ‘non-restorative sleep’ (i.e. feeling not refreshed when waking up) improved among participants in the exercise condition (results can be obtained from the first author). Contrary to expectations, we did not find an effect of exercise on sleep duration. This results may be explained by the fact that, at pre-intervention, 82.83% of the students already slept between the 7–9 hours that are recommended for young adults of 18–25 years of age [67], which left not that much potential for improvement.

Based on the idea that exercise generates feelings of personal mastery, we expected the exercise intervention to increase participants’ self-efficacy. However, no such effect was found. One explanation for this non-significant finding may be that—at least during the relatively short duration of the intervention period—exercise does not benefit self-efficacy in general, but rather affects participants’ ‘exercise self-efficacy’. This type of self-efficacy specifically refers to confidence in one’s ability to exercise on a regular basis and has indeed been found to be improved after exercise interventions [30].

We did not find the expected improvement in physical fitness among students who received the exercise intervention. It is possible that the intensity of running in this study was too low to induce substantial changes in VO_2_max. Indeed, a previous study by Puetz, Flowers and O’Connor [23] also failed to find changes in VO_2_max in fatigued students who received low intensity exercise for six weeks, but did find such changes among students who received moderate intensity exercise for six weeks [23]. Yet another possibility is that the measurement of physical fitness, which was based on the Conconi paradigm, lacks validity [47–49]. At least, our results seem to illustrate that a change in VO_2_max is no precondition for fatigue to reduce, as is basically suggested by the cardiovascular fitness hypothesis [68]. We did find that more time spent on exercise during the follow-up period was related to higher VO_2_max at the 12-week follow-up.

The exercise intervention under study was effective in improving some of the indicators of cognitive functioning. Exercisers showed a larger improvement in self-reported cognitive functioning in daily life and a larger decrease in how demanding they experienced the cognitive tests as compared to controls. This latter decrease could be attributed to the lower fatigue levels exercisers displayed after the intervention period [58]. We also found a larger improvement in objectively measured inhibition in the exercise group. This finding should be interpreted with caution, though, since it could also be attributed to baseline differences between the two conditions and a tendency toward the mean. Thus, although an improvement in self-reported cognitive functioning was found, this result did only slightly co-occur with changes in objective cognitive performance. A certain dissimilarity between self-reported and objectively measures of indicators of cognition has also been found in previous research [50,56], and clear explanations have not yet been provided. Overall, the results of the current study are in line with earlier meta-analyses demonstrating inconclusive results of exercise on cognitive performance [69,70]. To develop a full picture of the relation between exercise and cognitive functioning, additional studies will be needed. For instance it is still not known which type, intensity or frequency of exercise is best for optimal effects on cognition [70].

Regarding the third aim of this study, we indeed found that the beneficial effects of the intervention were maintained at 4 and 12 weeks after the intervention. Additionally, emotional exhaustion, overall fatigue, and sleep quality had further improved 12 weeks after the intervention. These improvements were not affected by the amount of exercise participants engaged in during the follow up period. This might be due to lack of variance in time spent on exercising, as most exercisers chose to engage in exercise after the intervention period (80%) on a regular basis (113.93 minutes [*SD* = 88.51] a week).

### Strengths, limitations, and suggestions for future research

We believe that a major strength of this study is its RCT-design, including longitudinal follow-up measures, and intention-to-treat analysis [71]. Moreover, we used validated self-reports as well as objective cognitive performance measures asd, and we collected physiological data (physical fitness test) to obtain a more complete picture of the effects of the intervention. Future research may extend this approach by using other objective measures, such as sleep monitoring [72] or cortisol sampling [73], to gain more in depth knowledge about the (psychophysiological) effects of exercise on study-related fatigue.

On a more practical note, another strength may well be our intervention program. We found that low intensity running three times a week had beneficial effects on various outcomes. Although it remains unknown whether this dose/type of exercise delivers optimal effects on the study outcomes, we showed that this intervention is at least feasible and accessible for university students with high levels of study-related fatigue, as dropout remained very low (i.e., 8% drop out rate) and compliance was high (i.e., 81% of running sessions was attended). Furthermore, a large majority of the students (i.e., 80%) chose to still engage in regular exercise in the 12 weeks after the intervention. It thus seems that this intervention has the potential to stimulate regular exercise patterns in the longer term. Future studies are recommended in which different exercise doses or types can be compared for investigating optimal effects on our outcomes. We believe that the exercise intensity in such studies should not be too high, as this may not or even negatively impact outcomes such as fatigue [74], cognitive performance [75], and sleep quality [27], especially in participants who are already fatigued at the start.

Apart from these strengths, several theoretical and methodological issues deserve discussion. These relate to the choice of employing a non-blinded wait-list design. As a result of using a wait list as control condition, we cannot rule out that the positive study findings are (partly) due to other ingredients of the intervention than exercise itself (i.e., non-specific factors), such as social support that may be provided in the group running sessions. Another consequence of this design is that we could not make firm conclusions about follow-up effects, as it was not possible to compare exercisers and controls in that period (i.e., controls received the exercise intervention after six weeks of waiting). Despite the limitations associated with a wait list design, we believe the choice for such a design is justified, because it is suitable for a first evaluation of a novel intervention [76] and because a proven effective standard intervention to reduce study-related fatigue does not exist yet. Another potential limitation of the chosen design is lack of blinding, which may have enhanced the possibility that positive expectations of the participants influenced the results [77]. It should be noted, though, that blinding of the participants was by definition not possible, because they received an ‘active’ intervention. Researchers and trainers in the current study were not blinded as well, since practical issues relating to the wait list design did not allow us to do so. However, as our measures did not involve subjective evaluations by the researchers involved in the study, we believe our study’s findings were not biased by lack of blinding. We recommend that future randomized controlled trials employ a design and measurements that make it possible to further distillate whether specific (i.e., exercise) and/or non-specific factors (i.e., social support, placebo effect) of the intervention are responsible for the beneficial effects on study-related fatigue. For instance, a comparison between individual versus group exercise may reveal to what extent social support is responsible for positive effects.

### Theoretical and practical contributions

We believe the results of the present study contribute to current evidence about exercise and study-related fatigue both theoretically and practically. With respect to theoretical contributions, we were among the first to investigate the association between exercise and study-related fatigue by using a strong methodological design. We showed that regular exercise at a low intensity has major benefits compared to time alone. Moreover, these effects did not fade away and persisted till at least twelve weeks after the intervention. Results found in this current study could form a basis for future studies investigating the working mechanisms of exercise on study-related fatigue and effectiveness studies that examine the effects of the intervention in daily practice.

With regard to practical contributions, we showed that exercise can effectively be applied as an intervention for student well-being. Furthermore, follow-up measures imply that the intervention has the potential to promote regular exercise and accompanying beneficial effects in the longer run. As exercise is accessible, simple, and inexpensive, this study offers practical suggestions for students and professionals who work with students. Students experiencing fatigue problems should be supported and encouraged to engage in regular exercise, for instance by offering a university exercise programs like the exercise intervention under study.

## Supporting Information

S1 CONSORT Checklist (DOC)

S1 Protocol Approval Ethics Committee (in Dutch). (PDF)

S2 Protocol Application form Ethics Committee (in English). (DOCX)

## References

[1] Lingard H. Conflict between paid work and study: does it impact upon student’s burnout and satisfaction with university life? J Educ Built Env. 2007; 2, 90–109.

[2] Schaufeli WB, Martínez IM, Marques Pinto A, Salanova M, Bakker AB. Burnout and engagement in university students: a cross-national study. J Cross Cult Psychol. 2002; 33: 464–481.

[3] Balogun JA, Hoeberlein-Miller TM, Schneider E, Katz JS. Academic performance is not a viable determinant of physical therapy students’ burnout. Percept Motor Skill. 1996; 83: 21–22. 10.2466/pms.1996.83.1.21 8873168

[4] Jacobs SR, Dodd D. Student burnout as a function of personality, social support, and workload. J Coll Stud Dev. 2003; 44: 291–303.

[5] Law DW. Exhaustion in university students and the effect of coursework involvement. J Am Coll Health. 2007; 55, 239–245. 17319330 10.3200/JACH.55.4.239-245

[6] Schmidt E, Simons M. Psychologische klachten onder studenten [psychological problems among students]. Landelijke Studenten Vakbond [National Students Union]. 2015. Available: http://www.lsvb.nl/actueel/persbericht/psychische-klachten-bij-helft-studenten

[7] Mailey EL, Wojcicki TR, Motl RW, Hu L, Strauser DR, Collins KD et al Internet-delivered physical activity intervention for college students with mental health disorders: a randomized pilot trial. Psychol Health Med. 2010; 15: 646–659. 10.1080/13548506.2010.498894 21154018

[8] Vossensteyn H, Cremonini L, Epping E, Laudel G, Leisyte L. International experiences with student financing: tuition fees and student financial support in perspective [Report]. 2013. Available: https://www.rijksoverheid.nl/documenten/rapporten/2013/03/31/international-experiences-with-student-financing-tuition-fees-and-student-financial-support-in-perspective

[9] Maslach C, Schaufeli WB, Leiter MP. Job Burnout. Annu Rev Psychol. 2001; 52: 397–422. 11148311 10.1146/annurev.psych.52.1.397

[10] Campos JADB, Jordani PC, Zucoloto ML, Bonafé FSS, Maroco J. Burnout syndrome among dental students. Rev Bras Epdemiol. 2012; 15, 155–165. 10.1590/s1415-790x2012000100014 22450501

[11] Bernaards CM, Jans MP, Van den Heuvel SG, Hendriksen IJ, Houtman IL, Bongers PM. Can strenuous leisure time physical activity prevent psychological complaints in a working population? Occup Environ Med. 2006; 63: 10–16. 16361400 10.1136/oem.2004.017541 PMC2078023

[12] Carson RL, Baumgartner JJ, Matthews RA, Tsouloupas CN. Emotional exhaustion, absenteeism, and turnover intentions in childcare teachers: examining the impact of physical activity behaviors. J Health Psychol. 2010; 15: 905–914. 10.1177/1359105309360697 20472609

[13] Gerber M, Brand S, Elliot C, Holsboer-Trachsler E, Pühse U, Beck J. Aerobic exercise training and burnout: a pilot study with male participants suffering from burnout. BMC Res Notes. 2013; 6: 78 10.1186/1756-0500-6-78 23497731 PMC3599602

[14] Jonsdottir IH, Rödjer L, Hadzibajramovic E, Börjesson M, Ahlborg G. A prospective study of leisure-time physical activity and mental health in Swedish health care workers and social insurance officers. Prev Med. 2010; 51: 373–377. 10.1016/j.ypmed.2010.07.019 20691721

[15] Otto M, Smits JAJ. Exercise for mood and anxiety. Oxford: University Press; 2011.

[16] Sonnentag S. Psychological detachment from work during leisure time. Curr Dir Psychol Sci. 2012; 21: 114–118.

[17] Geurts S. (2014). Recovery from work during off-job time In: Bauer G. & Hämmig O. (Eds.). Bridging occupational, organizational and public health. A transdisciplinary approach (pp.193–208). Dordrecht: Springer Science + Business Media.

[18] Geurts SAE, Sonnentag S. Recovery as an explanatory mechanism in the relation between acute stress reactions and chronic health impairment. Scand J Work Environ Health. 2006; 32: 482–492. 17173204 10.5271/sjweh.1053

[19] Rook JW, Zijlstra FRH. The contribution of various types of activities to recovery. Eur J Work Organ Psychol. 2006; 15: 218–240.

[20] Dishman RK, Jackson EM. Cardiorespiratory fitness and laboratory stress: a meta-regression analysis. Psychophysiol.2006; 43: 57–72. 10.1111/j.1469-8986.2006.00373.x 16629686

[21] Salmon P. Effects of physical exercise on anxiety, depression, and sensitivity to stress: a unifying theory. Clin Psychol Rev. 2001; 21, 33–61. 11148895 10.1016/s0272-7358(99)00032-x

[22] Gerber M, Brand S, Elliot C, Holsboer-Trachsler E, Pühse U, Beck J. Aerobic exercise training and burnout: a pilot study with male participants suffering from burnout. BMC Res Notes, 2013; 6: 78 10.1186/1756-0500-6-78 23497731 PMC3599602

[23] Puetz TW, Flowers SS, O’Connor PJ. A randomized controlled trial of the effect of aerobic exercise training on feelings of energy and fatigue in sedentary young adults with persistent fatigue. Psychother Psychosom. 2008; 77: 167–174. 10.1159/000116610 18277063

[24] Pagnin D, de Queiroz V, Carvalho YT, Dutra AS, Amaral MB, Queiroz TT. The relations between burnout and sleep disorders in medical students. Acad Psychiatry 2014, 38: 438–444. 10.1007/s40596-014-0093-z 24683060

[25] Ekstedt M, Söderström M Akerstedt T, Nilsson J, Søndergaard HP, Aleksander P. Disturbed sleep and fatigue in occupational burnout. Scand J Work Environ Health. 2006; 32: 121–131. 16680382 10.5271/sjweh.987

[26] Curcio G, Ferrara M, De Gennaro L. Sleep loss, learning capacity and academic performance. Sleep Med Rev. 2006; 10, 323–337. 16564189 10.1016/j.smrv.2005.11.001

[27] Driver H, Taylor SR. Exercise and sleep. Sleep Medicine Reviews 2000, 4: 387–402. 12531177 10.1053/smrv.2000.0110

[28] Shoji K, Cieslak R, Smoktunowicz E, Rogala A, Benight CC, Luszczynska A. Associations between job burnout and self-efficacy: a meta-analysis. Anxiety Stress Coping. 2015; 14, 1–20. 10.1080/10615806.2015.1058369 26080024

[29] Craft LL. Exercise and clinical depression: examining two psychological mechanisms. Psychol Sport Exerc. 2005; 6:151–171.

[30] McAuley E, Mailey EL, Szabo AN, Gothe N. Physical activity and personal agency: self-efficacy as a determinant, consequence, and mediator In: Routledge Handbook of Physical Activity and Mental Health. Ekkekakis (ed.). Abingdon: Routledge; 2013 p. 224–235.

[31] Haskell WL, Lee IM, Pate RR, Powell KE, Blair SN, Franklin BA, et al Physical activity and public health: updated recommendation for adults from the American College of Sports Medicine and the American Heart Association. Med Sci Sports Exerc. 2007; 39: 1423–1434. 17762377 10.1249/mss.0b013e3180616b27

[32] Deligkaris P, Panagopoulou E, Montgomery AJ, Masoura E. Job burnout and cognitive functioning: a systematic review. Work Stress. 2014; 28: 107–123.

[33] Hillman CH, Erickson KI, Kramer AF. Be smart, exercise your heart: exercise effects on brain and cognition. Nat Rev Neurosci. 2008; 9: 58–65. 18094706 10.1038/nrn2298

[34] Van Praag H. Neurogenesis and exercise: past and future directions. Neuromol Med. 2008; 10: 128–140. 10.1007/s12017-008-8028-z 18286389

[35] Schaufeli WB, Van Dierendonck D. Utrechtse Burnout Schaal (UBOS). [The Utrecht Burout Scale (UBOS)] De Psycholoog. 2001; 36:9–12

[36] De Vries J, Michielsen H, Van Heck GL, Drent M. Measuring fatigue in sarcoidosis: the Fatigue Assessment Scale (FAS). Br J Health Psychol. 2004; 9: 279–291. 15296678 10.1348/1359107041557048

[37] Persinger R, Foster C, Gibson M, Fater DC, Porcari JP. Consistency of the talk test for exercise prescription. Med Sci Sport Exerc. 2004; 36: 1632–1626. 15354048

[38] Loy BD, O’Connor PJ, Dishman RK. The effect of a single bout of exercise on energy and fatigue states: a systematic review and meta-analysis. Fatigue Biomed Health Beh. 2013: 1: 223–242.

[39] Hreljac A. Impact and overuse injuries in runners. Med Sci Sports Exerc. 2004; 36, 845–849. 15126720 10.1249/01.mss.0000126803.66636.dd

[40] Wenger HA, Bell GJ. The interactions of intensity, frequency and duration of exercise training in altering cardiorespiratory fitness. Sports Med. 1986; 3: 356–356. 10.2165/00007256-198603050-00004 3529283

[41] Chan RCK, Shum D, Toulopoulou T, Chen EYH. Assessment of executive functions: review of instruments and identification of critical issues. Arch Clinc Neuropsychol. 2008; 23: 201–216. 10.1016/j.acn.2007.08.010 18096360

[42] Maslach C., Jackson S.E. and Leiter M.P. (1996), MBI: The Maslach Burnout Inventory: Manual, Consulting Psychologists Press, Palo Alto, CA.

[43] Van Veldhoven M, Broersen S. Measurement quality and validity of the “need for recovery scale”. Occup Environ Med. 2003; 60, i3–i9. 12782740 10.1136/oem.60.suppl_1.i3 PMC1765728

[44] Van Veldhoven M, Meijman T. Het meten van psychosociale arbeidsbelasting met een vragenlijst: de vragenlijst beleving en beoordeling van de arbeid (VBBA) [The measurement of psychosocial workload with the help of a questionnaire: the questionnaire of experience and evaluation of work]. Amsterdam: Nederlands Instituut voor Arbeidsomstandigheden; 1994.

[45] Roth T. Insomnia: definition, prevalence, etiology, and consequences. J Clin Sleep Med. 2007; 3: S7–S10. 17824495 PMC1978319

[46] Bosscher RJ, Smit JH. Confirmatory factor analysis of the General Self-Efficacy Scale. Beh Res Ther. 1998; 36, 339–343. 10.1016/s0005-7967(98)00025-4 9642852

[47] Conconi F, Grazzi G, Casoni I, Guglielmini C, Borsetto C, Ballarin E, et al The Conconi test: Methodology after 12 years of application. Int J Sports Med. 1996; 17, 509–519. 8912066 10.1055/s-2007-972887

[48] Bosquet L, Léger L, Legros P. Methods to determine aerobic endurance. Sports Med. 2002; 32: 675–700. 12196030 10.2165/00007256-200232110-00002

[49] Bodner ME, Rhodes E. A review of the concept of the heart rate deflection point. Sports Med. 2000; 30:41–46. 10.2165/00007256-200030010-00004 10907756

[50] Oosterholt BG, Van der Linden D, Maes JHR, Verbraak MJPM, Kompier MAJ. Burned out cognition—cognitive functioning of burnout patients before and after a period with psychological treatment. Scand J Work Environ Health. 2012; 38, 358–369. 10.5271/sjweh.3256 22025205

[51] Broadbent DE, Cooper PF, FitzGerald P, Parkes KR. The Cognitive Failures Questionnaire (CFQ) and its correlates. Br J Clin Psychol. 1982; 21: 1–16. 7126941 10.1111/j.2044-8260.1982.tb01421.x

[52] Miyake A, Friedman NP, Emerson MJ, Witzki AH, Howerter A, Wager TD. The unity and diversity of executive functions and their contributions to complex “Frontal Lobe” tasks: a latent variable analysis. Cogn Psychol. 2000; 41: 49–100. 10945922 10.1006/cogp.1999.0734

[53] Kirchner WK. Age differences in short-term retention of rapidly changing information. J Exp Psychol. 1958;55:352–8. 13539317 10.1037/h0043688

[54] Robertson IH, Manly T, Andrade J, Baddeley BT, Yiend J. ‘Oops!’: Performance correlates of everyday attentional failures in traumatic brain injured and normal subjects. Neuropsychologia. 1997; 35: 747–58. 9204482 10.1016/s0028-3932(97)00015-8

[55] Poljac E, Simon S, Ringlever L, Kalcik D, Groen WB, Buitelaar JK, et al Impaired task switching performance in children with dyslexia but not in children with autism. Q J Exp Psychol. 2010; 63:401–16. 10.1080/17470210902990803 19565430

[56] Oosterholt BG, Meas JH, Van der Linden D, Verbraak MJ, Kompier MAJ. Cognitive performance in both clinical and non-clinical burnout. Stress, 2014 17: 400–409. 10.3109/10253890.2014.949668 25089935

[57] Hockey RV. The psychology of fatigue: work, effort and control. Cambridge University Press, Cambridge; 2013.

[58] Meijman TF, Mulder G. Arbeidspsychologische aspecten van werkbelasting. [Workpsychological aspects of work-demands] In: Drenth PJD, Thierry H, De Wolf CJ, editors. Handboek A&O psychologie [Handbook Work & Organizational Psychology]. Deventer: Van Loghum Slaterus; 1992.

[59] Faul F, Erdfelder E, Lang AG, Buchner A. G*Power 3: a flexible statistical power analysis program for the social, behavioral, and biomedical sciences. Behav Res Methods. 2007; 39: 175–191. 17695343 10.3758/bf03193146

[60] IBM SPSS Statistics for Windows, Version 19.0. Armonk, NY: IBM Corp 2010

[61] Moher D, Schulz KF, Altman DG. The CONSORT statement: revised recommendations for improving the quality of reports of parallel group randomized trials. BMC Med Res Methodol. 2001; 1: 2 11336663 10.1186/1471-2288-1-2 PMC32201

[62] Jacobson NS, Truax P. Clinical significance: a statistical approach to defining meaningful change in psychotherapy research. J Consult Clin Psych. 1991; 59: 12–19. 10.1037//0022-006x.59.1.12 2002127

[63] Ferguson CJ. An effect size primer: a guidelines for clinicians and researchers. Prof Psychol Res Pr. 2009; 40, 532–538.

[64] Abdi H. Bonferroni and Sidak corrections for multiple comparisons. Encyclopedia of Measurement and Statistics. 2007.

[65] Cohen J. Statistical power analysis for the behaviorial sciences. Mahwah: Erlbaum; 1988.

[66] Larun L, Brurberg KG, Odgaard-Jensen J, Price JR. Exercise therapy for chronic fatigue syndrome. Cochrane Database Syst. Rev. 2015: CD003200 10.1002/14651858.CD003200.pub3 25674924

[67] Hirshkowitz M, Whiton K, Albert SM, Alessi C, Bruni O, DonCarlos L. National Sleep Foundation’s sleep time duration recommendations: methodology and results summary. Sleep Health. 2015; 1: 40–43. 29073412 10.1016/j.sleh.2014.12.010

[68] Colcombe S, Kramer AF. Fitness effects on the cognitive function of older adults: a meta-analytic study. Psychol Sci. 2003; 14: 125–130. 12661673 10.1111/1467-9280.t01-1-01430

[69] Smith PJ, Blumenthal JA, Hoffman BM, Cooper H, Strauman TA, Welsh-Bohmer K, Browndyke JN et al Aerobic exercise and neurocognitive performance: a meta-analytic review of randomized controlled trials. Psychosom Med. 2010; 72: 239–252. 10.1097/PSY.0b013e3181d14633 20223924 PMC2897704

[70] Verburgh L, Königs M, Scherder EJ, Oosterlaan J. Physical exercise and executive functions in preadolescent children, adolescents and young adults: a meta-analysis. Br J Sports Med. 2014; 48: 973–979. 10.1136/bjsports-2012-091441 23467962

[71] Sibbald B, Roland M. Understanding controlled trials: Why are randomized controlled trials important? BMJ. 1998; 316: 201 9468688 10.1136/bmj.316.7126.201 PMC2665449

[72] Van de Water AT, Holmes A, Hurley DA. Objective measurements of sleep for non-laboratory settings as alternatives to polysomnography—a systematic review. J Sleep Res. 2011; 20: 183–200. 10.1111/j.1365-2869.2009.00814.x 20374444

[73] Oosterholt BG, Maes JH, Van der Linden D, Verbraak MJ, Kompier MAJ. Burnout and cortisol: evidence for a lower cortisol awakening response in both clinical and non-clinical burnout. J Psychosom Res. 2015; 78: 445–451. 10.1016/j.jpsychores.2014.11.003 25433974

[74] Brooks KA, Carter JG. Overtraining, exercise, and adrenal insufficiency. J Nov Physiother. 2013; 3: 11717 23667795 10.4172/2165-7025.1000125

[75] Chang YK, Labban JD, Gapin JI, Etnier JL. The effects of acute exercise on cognitive performance: a meta-analysis. Brain Res. 2012; 1453: 87–201. 10.1016/j.brainres.2012.02.068 22480735

[76] Mohr D, Spring B, Freedland KE, Beckner V, Arean P, Hollon SD et al The selection and design of control conditions for randomized controlled trials of psychological interventions. Psychother Psychosom. 2009; 78: 275–284. 10.1159/000228248 19602916

[77] Lindheimer JB, O’Connor PJ, Dishman RK. Quantifying the placebo effect in psychological outcomes of exercise training: a meta-analysis of randomized trials. Sports Med. 2015; 45: 693–711. 10.1007/s40279-015-0303-1 25762083

