# Supplementary material for: Exercise as an Intervention to Reduce Study-Related Fatigue among University Students: A Two-Arm Parallel Randomized Controlled Trial
Source: PLoS One. 2016 Mar 31;11(3):e0152137. doi: 10.1371/journal.pone.0152137 (PMC4816334; doi:10.1371/journal.pone.0152137)
Supplement: S1 Protocol — (PDF) [file pone.0152137.s002.pdf]

A

Faculteit der Sociale Wetenschappen

Montessorilaan 3
Postbus 9104
6500 HE Nijmegen

Telefoon +31 24 36 16236
Fax +31 24 36 11798

www.ru.nl/fsw

Prof. dr. A. Cillessen
Directeur BSI a.i.
Montessorilaan 3
INTERN

Ons kenmerk	Uw kenmerk	Telefoon	Datum
OOM/MB/13U.016206		024-3616236	29 november 2013

Betreft	E-mail
toetsing ECSW2013-1811-142 onderzoek De Vries/Kompier	m.blijleven@socsci.ru.nl

90 YEAR ANNIVERSARY

Geachte directeur,

Met de bijgesloten notitie heeft de voorzitter van de facultaire Ethiek Commissie (ECSW), prof. dr. G. van Luijtelaar, het faculteitsbestuur op de hoogte gesteld van het advies van de commissie over het onderzoek van mw. J. de Vries MSc en prof. dr. M. Kompier: *Effect of exercise on fatigue among students* (zie bijlage).

Wij kunnen u meedelen dat het faculteitsbestuur het positieve advies van de commissie integraal overneemt.

Met vriendelijke groet,

prof. dr. D.H.J. Wigboldus
decaan

mr. A.H.M. van Berkum
secretaris

bijlage(n): 1
cc: -prof. dr. G. van Luijtelaar
-prof. dr. M. Kompier
-mw. J. de Vries MSc

ARCHIEFCOPIE
CLASS.no. ..07.58.

Faculteit der Sociale
Wetenschappen
Postbus 9104
6500 HE Nijmegen
Telefoon 024 36 16237
Fax 024 36 11798
www.ru.nl/fsw

Ethiek Commissie (ECSW)
Faculteit der Sociale Wetenschappen

Betreft:	aanmelding ECSW2013-1811-142 De Vries/Kompier; *Effect of exercise on fatigue among students*
Van:	prof. dr. G. van Luijtelaar, voorzitter ECSW
Cc.:	mr. M. Blijleven, secretaris ECSW
Datum:	28 november 2013
Status:	ADVIES

Na kennisname van het aanmeldingsformulier van mw. J. de Vries MSc en prof. dr. M. Kompier met betrekking tot het onderzoek:

Effect of exercise on fatigue among students,

is de facultaire Ethiek Commissie (ECSW) op 18 november 2013 tot het volgende oordeel gekomen:

de commissie heeft geen bezwaar tegen dit onderzoek.

Met vriendelijke groet,

prof. dr. G. van Luijtelaar,
voorzitter Ethiek Commissie
Faculteit Sociale Wetenschappen, RU Nijmegen.

Bedankt voor het invullen. Het formulier is verstuurd naar de ECG. Bewaar of print deze pagina voor uw eigen administratie. Vergeet niet de ontbrekende documenten te mailen naar m.blijleven@socsci.ru.nl.

AANMELDINGSFORMULIER ECG

I. ALGEMENE RICHTLIJNEN BIJ HET GEBRUIK VAN DIT FORMULIER

Dit formulier moet voor elk onderzoek worden ingevuld en ingediend bij de facultaire Ethische Commissie Gedragswetenschappelijk onderzoek (ECG). Alleen als er een toelichting gegeven wordt zal de ECG in een vergadering een advies formuleren.

1. Dit formulier kan gebruikt worden voor een individueel experiment (project) of voor een onderzoekslijn. Onderzoekers worden aangemoedigd onderzoekslijnen in te dienen, waarin meerdere experimenten worden voorgesteld die inhoudelijk samenhangen en waarin dezelfde procedures en populaties worden gebruikt. Alleen eerstverantwoordelijke onderzoekers kunnen een protocol indienen, via hun email adres aan de Universiteit te Nijmegen.
2. Ethische goedkeuring van een project of onderzoekslijn is geldig voor een periode van 5 jaar of tot er zich een wijziging voordoet in de hieronder verschafte informatie. Onderzoekers moeten jaarlijks de ECG informeren of het ingediende protocol nog actief is. Wanneer een project of onderzoekslijn is beëindigd, moet de ECG hierover onmiddellijk ingelicht worden.
3. De hieronder vermelde onderzoeker en medewerkers verbinden er zich toe proefpersonen te behandelen volgens de principes van de verklaring van Helsinki (zie ECG-website), en te garanderen dat proefpersonen (wilsbekwaam of –onbekwaam, in een afhankelijkheidsrelatie tot de onderzoeker of niet) te allen tijde medewerking kunnen opzeggen zonder enige gevolgen.
4. De onderzoeker verbindt zich tot het maximaliseren van de kwaliteit van het onderzoek, de statistische analyse en rapportering, en tot het respecteren van specifieke regelgeving in verband met specifieke methodes (vb. fMRI).
5. De onderzoeker verklaart het onderzoek naar waarheid beschreven te hebben, met bijzondere aandacht voor ethische aspecten.

Voor akkoord (Naam in hoofdletters)

hoogleraar	Michiel Kompier
directeur onderzoeksinstituut	Toon Cillissen
datum	01Nov2013

II. ALGEMENE INFORMATIE

Titel	Effect of exercise on fatigue among students
Subsidieverstrekker	1e geldstroom
Naam projectbegeleider	Michiel Kompier
E-mail adres projectbegeleider	m.kompier@psych.ru.nl
Naam en projectuitvoerder	J.D. de Vries
E-mail adres projectuitvoerder	j.devries@psych.ru.nl
Locatie(s) van het onderzoek	Radboud Universiteit Nijmegen

Vat hieronder het onderzoek samen (maximaal 500 woorden, inclusief beschrijving van procedure, methode en groep proefpersonen). Indien van toepassing, verstrek voldoende informatie over het gebruik van ethisch gevoelig liggende manipulaties of stimulusmateriaal. Samenvatting
Research indicates that full time students can experience high levels of fatigue or even burn-out problems (Balogun et al., 1996). According to Mailey et al. (2010) the prevalence of (study-related) fatigue among college students is rising.
The aim of the current study is to find out whether an exercise intervention has positive effects in terms of improved physical fitness [Hypothesis 1], reduced fatigue problems [H2], improved levels of general health and well-being [H3], cognitive functioning [H4], self-efficacy [H5], and participation in daily life [H6] in a group of university students who suffer from high levels of fatigue.

Sample and Design
This study will be conducted among 120 university students (in Nijmegen) with relatively high levels of fatigue. We will use an experimental design in which participants will be randomly allocated to either a 6

-week exercise condition (experimental condition, n=60) or a control group (waitlist condition in which the students will receive the exercise intervention after they have served as a control group for 6 weeks, n=60). The exercise intervention will cover a 6-week period in which the participant will run under supervision of a licensed running trainer twice a week, and independently once a week. Exercise intensity will be set at approximately 70% of VO2-max.
Participants will be excluded when they are 1) currently exercising more than one hour a week; 2) dependent on alcohol/drugs; 3) currently or in the previous two months using medications that are of influence on mood; 4) currently having, the previous half year had, or currently on a waitinglist for psychological/medical treatment; 5) have a medical cause for their fatigue; 6) having a medical contra-indication for physical activity

Intensive repeated measures will be collected for all participants:
-Pre: Once before the intervention: pre-screening on T1
-Inter: Six times during the intervention: T2 to T7 (i.e. every week during the 6 week period)
-After: Three times after the intervention: two weeks after the intervention (T8), four weeks after the intervention (T9), and 12 weeks after the intervention (T10).

Variables
Physical fitness
-Assessed by a graded exercise test (performed on a stationary bike), whereby participants exercise to around 85% of maximal heart rate. We will follow the procedure comparable to that in Blumenthal et al. (2007). The exercise will be conducted at the begin of the program (T1). The same assessment will be used at the conclusion (T7) and at follow-up of the intervention (T10).
Fatigue/exhaustion
-The severity of fatigue symptoms will be measured with a modified version of the Utrechtse Burn-Out Scale (UBOS; Schaufeli & Van Dierendonck, 2000), a Dutch adaptation of the Maslach Burn-out Inventory (MBI; Maslach et al., 1996). It will be adapted for use in student samples, following Schaufeli et al. (2002). From this questionnaire we will use the scale Exhaustion (5 items). Items are scored on a 7-point frequency rating scale (0 = "never", 6 = "every day"). High scores are indicative for high levels of fatigue. We will use cut off scores that have been developed by Schaufeli and van Dierendonck (2000). Measured at T1 to T10.
-Need for recovery will be assessed with the 6-item 'need for recovery scale' (Van Veldhoven et al., 2002). Measured at T1 to T10.
-Fatigue will be measured by using the 10-item Fatigue Assessment Scale (FAS) developed and validated by Michielsen et al. (2003). Measured at T1 to T10.
Health and well-being
-Health & Well-being: Following De Bloom et al. (2010) we will employ seven single-item measures to tap several main indicators of health and well-being: health status, mood, stress, fatigue, tension, energy level and satisfaction. Participants will report with a report mark between 1 and 10.
-Sleep quality: five-items sleep quality scale (Van Veldhoven et al., 2002). (e.g. 'Last night I woke up several times'; 1= 'yes', 0 = 'no'). Measured at T1 to T10.
Cognitive functioning
-Executive functioning (working memory, inhibition, task switching) will be assessed by using the following measures: 2-Back task (Kirchner, 1958), the Sustained Attention to Response Test (SART) (Robertson et al., 1997) and the Matching task (Poljac et al., 2010). These are three well-validated tests, each tapping specifically into one of the three target functions (Oosterholt et al., in press). These tests will be assessed two times: at T1 and T7 (because of a possible learning effect).
-CFQ (Broadbent et al., 1982: Cognitive Failures Questionnaire). A Dutch translation (25 items) will be used to assess the participants' self-reported cognitive functioning in daily life. Measured at T1 and T7.
Self-efficacy
-Self-efficacy will be measured by the Dutch Self-Efficacy Scale (10 items, Schwarzer & Jerusalem, 1995) and one item report mark grade (on a scale from 1-10) provided by the participants (see Van Hooff et al., 2007). Measured at T1 to T10.
Participation in daily life
-We will also monitor participation in daily life (social interaction with family, friends, students, student networks). Measured at T1 to T10.
Control variables
-Exercise other than during the running of the exercise intervention. We will measure exercise activities as well as associated exercise experiences (pleasure and effort). Measured at T1 to T10.

Heeft u dit of soortgelijk onderzoek al eerder ingediend bij de ECG?

(x) Nee
( ) Ja, ECG-nummer

Betreft dit een onderzoeksproject of een onderzoekslijn? Onderzoeksproject

III. ETHISCHE RICHTLIJNEN

1. Hieronder staan een aantal regels waaraan volgens de ECG voldaan moet worden. Het navolgen van deze regels is de verantwoordelijkheid van de individuele onderzoeker of diens supervisor. Wordt aan de onderstaande regels (A -D) voldaan?

A. Het onderzoek zal naar verwachting nieuwe en belangrijke inzichten opleveren.
B. Het onderzoek voldoet aan de eisen van juiste methodologie.
C. Het onderzoek geschiedt onder supervisie van een deskundig persoon. Diegene die mogelijke ingrepen verricht dient deskundig en ervaren te zijn.
D. Het onderzoek vindt niet plaats met proefpersonen die buiten het onderzoek in een ondergeschikte positie ten aanzien van de onderzoeker verkeren (bv. eigen kinderen).

(x) Ja
( ) Nee, toelichting:

2. Is er een aanspreekpunt waar proefpersonen terecht kunnen met vragen over het onderzoek en worden zij hiervan op de hoogte gesteld?

(x) Ja
( ) Nee, toelichting:

3. Is duidelijk waar klachten over deelname aan het onderzoek kunnen worden geuit en hoe deze behandeld zullen worden?

(x) Ja
( ) Nee, toelichting:

4. Zijn de proefpersonen volledig vrij om deel te nemen aan het onderzoek, en om hiermee op elk moment te stoppen wanneer zij dat willen, om welke reden dan ook?

(x) Ja
( ) Nee, toelichting:

5. Worden proefpersonen en/of hun vertegenwoordiger voor deelname voorgelicht over doel, aard en duur, risico's en bezwaren van de studie, en tekenen zij voor deelname middels de standaard informed consent (zie de formulieren op de website)?

Het is standaard dat proefpersonen of hun wettelijke vertegenwoordiger

a. vooraf schriftelijk en mondeling over de aard van het onderzoek volledig zijn ingelicht
b. schriftelijke toestemming geven via het gepaste Toestemmingsformulier
c. terugkoppeling: achteraf schriftelijk en mondeling in kennis worden gesteld van de doelstelling van het onderzoek

De voorlichting is duidelijk en afgestemd op de proefpersoon (bijvoorbeeld ingeval van minderjarigen). De proefpersoon krijgt bedenktijd tussen informatie en toestemming. Indien volledige informatie vooraf onmogelijk is vanwege de aard van het onderzoek, dient de proefpersoon achteraf te worden geïnformeerd. Ook minderjarige en wilsonbekwame proefpersonen moeten in alle gevallen toch naar bevattingsvermogen worden geïnformeerd.

(x) Ja
( ) Nee, toelichting:

6. Indien er misleiding plaatsvindt, voldoet de procedure dan aan de standaardeisen (geen misleiding over risico's, accurate debriefing)?

Onder misleiding wordt verstaan het verschaffen van inaccurate of onvolledige informatie aan de proefpersoon. Misleiding is slechts toegestaan als er geen mogelijkheid bestaat de vraagstelling zonder misleiding te beantwoorden. Misleiding is niet toegestaan als het gaat om informatie over

de eventuele risico's die verbonden zijn aan deelname. Na misleiding vindt er altijd een volledige debriefing van de proefpersoon plaats over de manier waarop de proefpersoon is misleid. Indien er redelijkerwijs tijdelijke negatieve effecten van een misleiding zijn te verwachten, dan vindt deze debriefing plaats onmiddellijk na het beëindigen van het experiment (bijvoorbeeld als er valse negatieve feedback wordt gegeven over intelligentiescores, dan vindt er onmiddellijke debriefing plaats). De debriefing is op zo'n manier gesteld dat redelijkerwijs mag worden verwacht dat de tijdelijke negatieve effecten op bijvoorbeeld zelfbeeld en stemming door de debriefing worden weggenomen. Indien geen tijdelijke negatieve effecten worden verwacht mag de debriefing ook op een later tijdstip plaatsvinden, echter uiterlijk binnen een maand na het beëindigen van het experiment.

(x) Er vindt geen misleiding plaats
( ) Er vindt misleiding plaats die geheel voldoet aan de eisen
( ) Er vindt misleiding plaats die niet voldoet aan de standaardeisen (toelichting)

7. Wordt voldaan aan de standaard regels in verband met anonimiteit en privacy?

De onderzoeksgegevens worden standaard

a. anoniem verwerkt en vertrouwelijk opgeslagen
b. met inzagerecht voor de proefpersoon van de eigen gegevens
c. met inzage van alle data voor alle onderzoekers betrokken bij het project

(x) Ja
( ) Nee, toelichting:

IV. DE PROEFPERSONEN

8. Gaat het om een gezonde populatie?

(x) Ja
( ) Nee, toelichting:
Het gaat om 'gezonde' proefpersonen, met vermoeidheidsklachten die geen klinische diagnose hebben (voor bijv. burnout of depressie), maar wel last hebben van vermoeidheidsklachten (relatief milde klachten dus). Deelnemers zijn fysiek gezond.

9. Worden de proefpersonen gescreend teneinde de risico's voor nadelige effecten van het onderzoek te reduceren?

(x) Nee
( ) Ja, toelichting:

10a. Is er sprake van onderzoek bij minderjarigen (<18 jaar) of bij wilsonbekwamen?

Wilsonbekwamen zijn personen die niet in staat zijn tot een redelijke waardering van hun belangen ter zake. (Zie de notitie 'nee tenzij')

( ) Ja, door met vraag 10b
(x) Nee, door met vraag 11

10b. Gaat het om doelgroepgebonden onderzoek?

Doelgroepgebonden onderzoek is onderzoek dat niet zonder deelname van de groep waartoe de proefpersoon behoort kan worden uitgevoerd, bv. minderjarigen, dementerende ouderen. (Zie de notitie 'nee tenzij')

( ) Ja
( ) Nee, toelichting:

10c. Is er een praktische reden waarom minderjarigen of wilsonbekwamen aan het onderzoek meedoen, bv. omdat er onvoldoende wilsbekwame personen geworven kunnen worden? (Zie de notitie 'nee tenzij')

( ) Nee
( ) Ja, toelichting:

10d. Is het mogelijk om de benodigde kennis eerst bij volwassen, wilsbekwame personen te verkrijgen?

(Zie de notitie 'nee tenzij')

( ) Nee
( ) Ja, toelichting:

10e. Is er sprake van therapeutisch onderzoek, d.w.z. dat de studie mede ten goede kan komen aan de proefpersoon zelf?

(Zie de notitie 'nee tenzij')

( ) Ja
( ) Nee, toelichting:

V. DE GEHANTEERDE METHODE

11. Wordt er een methode gebruikt die het mogelijk maakt bij toeval een bevinding te doen waarvan de proefpersoon op de hoogte zou moeten worden gesteld?

Sommige methoden van onderzoek kunnen toevalsbevindingen opleveren die van belang kunnen zijn voor de proefpersoon. Te denken valt aan hartritmestoornissen op een ECG, een afwijkend EEG (epilepsie), of een afwijking op een fMRI. Indien die mogelijkheid bestaat dient in het informed consent een bepaling te worden opgenomen die voorziet in de dan te volgen procedure. De proefpersoon dient bij die onderzoeken hetzij de naam en praktijkplaats van haar/zijn huisarts dan wel het complete praktijkadres van de huisarts of huisartsenpraktijk op te geven, die wordt verwittigd in het geval van een voor haar/hem van belang zijnde bevinding. Heeft de proefpersoon geen huisarts dan dient zij/hij akkoord te gaan met het feit dat de studentenarts, of in voorkomende gevallen een bedrijfsarts wordt verwittigd. De proefpersoon dient met deze procedure akkoord te gaan door middel van het ondertekenen van een aparte clausule op het informed consent formulier.

(x) Nee
( ) Ja, toelichting:

12. Wordt er gebruik gemaakt van unobtrusive methoden?

Bij unobtrusive methoden worden data verzameld, zonder dat de proefpersoon hiervan op de hoogte is. Het gedrag van de proefpersoon wordt bv. geobserveerd of op video opgenomen zonder dat deze het weet.

(x) Nee
( ) Ja, toelichting:

13. Worden proefpersonen aan handelingen onderworpen of wordt aan de proefpersonen een bepaalde gedragswijze opgelegd, die buiten de normale levenswijze vallen?

Voorbeelden: stress opwekken, druk uitoefenen om belangrijke normen en waarden te overschrijden, valse herinneringen induceren, verwerking van ernstige gebeurtenissen, langdurige of zeer frequente ondervraging, ambulante metingen, effectiviteit van interventies, onaangename psychische of fysieke symptomen oproepen binnen een experiment (Zie de notitie gedragswetenschappelijk onderzoek en de WMO)

(x) Nee
( ) Ja, toelichting:

De deelnemers aan het onderzoek wordt gevraagd om gedurende 6 weken 3x per week te gaan hardlopen. Twee keer per week onder leiding van een trainer en 1x voor zichzelf. De deelnemers beoefenen voor de start van de interventie (vrijwel) geen sport. De deelnemers zijn vrij om wel/niet 3x per week hard te lopen, maar het wordt wel aangemoedigd om deze 3x per week aan te houden. Na de interventie zijn de deelnemers vrij om het hardlopen voort te zetten of niet. Het zou mogelijk kunnen zijn dat deelnemers zich onprettig voelen als zij beginnen met de fysieke inspanning (bijv. snelle ademhaling, het gevoel geen lucht te krijgen, zware benen). De deelnemers worden twee keer begeleid door een opgeleide (hardloop)trainer (i.e. running therapeut) die kennis heeft van zowel het trainingsaspect als de psyche. De deelnemers wordt verteld om op zo'n tempo te lopen dat er nog gepraat kan worden (dus niet te hoge intensiteit).

14. Zijn de risico's en bezwaren minimaal? Is het risico dat men loopt een risico dat men in het dagelijks leven ook loopt?

(x) Ja
( ) Nee, toelichting:

15. Wordt er een andere dan de standaardvergoeding geboden aan de proefpersonen?

(x) Nee
( ) Ja, toelichting:

VI. AANVULLENDE INFORMATIE

Gebruik de hieronder voorziene ruimte voor aanvullingen of informatie waarvoor er op dit formulier onvoldoende ruimte voorzien was.

VII. TOEGEVOEGDE FORMULIEREN

Samen met dit formulier moeten de volgende documenten worden ingediend:

- de tekst van de advertenties;
- de tekst van de schriftelijke terugkoppeling (debriefing) met vermelding contactinformatie van verantwoordelijke onderzoeker;
- een kopie van het toestemmingsformulier;
- voor onderzoek op een externe locatie (buiten het Spinozagebouw), waar proefpersonen vallen onder de verantwoordelijkheid van een externe instelling (bv. school, ziekenhuis), moet een toestemmingsverklaring ondertekend door deze instelling bijgevoegd worden.

Deze documenten dient u zelf te mailen naar m.blijleven@socsci.ru.nl.

Vink aan welke documenten zijn toegevoegd:
[ ] Tekst van de advertenties
[ ] Schriftelijke terugkoppeling
[ ] Toestemmingsformulier proefpersoon.[1]

[1] Op de website vindt u een link met zogenaamde Toestemmingsformulieren. Deze formulieren kunt u zelf downloaden.
