# Supplementary material for: Exercise as an Intervention to Reduce Study-Related Fatigue among University Students: A Two-Arm Parallel Randomized Controlled Trial
Source: PLoS One. 2016 Mar 31;11(3):e0152137. doi: 10.1371/journal.pone.0152137 (PMC4816334; doi:10.1371/journal.pone.0152137)
Supplement: S2 Protocol — (DOCX) [file pone.0152137.s003.docx]

**Application form Ethics Committee** [Translation from Dutch to English]

**General information**

Title: Effect of exercise on fatigue among students

Supervisor project: Michiel Kompier ([m.kompier@psych.ru.nl](mailto:m.kompier@psych.ru.nl))

Name project coordinator: Juriena de Vries ([j.devries@psych.ru.nl](mailto:j.devries@psych.ru.nl))

Location research: Radboud University Nijmegen

**Summary research**

Research indicates that full time students can experience high levels of fatigue or even burn-out

problems (Balogun et al., 1996). According to Mailey et al. (2010) the prevalence of (study-related) fatigue among college students is rising. The aim of the current study is to find out whether an exercise intervention has positive effects in terms of improved physical fitness [Hypothesis 1], reduced fatigue problems [H2], improved levels of general health and well-being [H3], cognitive functioning [H4], self-efficacy [H5], and participation in daily life [H6] in a group of university students who suffer from high levels of fatigue.

*Sample and Design*

This study will be conducted among 120 university students (in Nijmegen) with relatively high levels of fatigue. We will use an experimental design in which participants will be randomly allocated to either a 6-week exercise condition (experimental condition, n=60) or a control group (waitlist condition in which the students will receive the exercise intervention after they have served as a control group for 6 weeks, n=60). The exercise intervention will cover a 6-week period in which the participant will run under supervision of a licensed running trainer twice a week, and independently once a week. Exercise intensity will be set at approximately 70oZo of V02-max. Participants will be excluded when they are 1) currently exercising more than one hour a week; 2) dependent on alcohol/drugs; 3) currently or in the previous two months using medications that are of influence on mood; 4) currently having, the previous half year had, or currently on a waiting list for psychological/medical treatment; 5) have a medical cause for their fatigue; 6) having a medical contraindication for physical activity

Intensive repeated measures will be collected for all participants:

-Pre: Once before the intervention: pre-screening on T1

-Inter: Six times during the intervention: T2 to T7 (i.e. every week during the 6 week period)

-After: Three times after the intervention: two weeks after the intervention (T8), four weeks after the intervention (T9), and 12 weeks after the intervention (T10).

*Variables*

Physical fitness

-Assessed by a graded exercise test (performed on a stationary bike), whereby participants exercise to around 8507o of maximal heart rate. We will follow the procedure comparable to that in Blumenthal et al. (2007). The exercise will be conducted at the begin of the program (T1). The same assessment will be used at the conclusion (T7) and at follow-up of the intervention (T10).

Fatigue/exhaustion

-The severity of fatigue symptoms will be measured with a modified version of the Utrechtse Burn-Out Scale (UBOS; Schaufeli S Van Dierendonck, 2000), a Dutch adaptation of the Maslach Burn-out Inventory (MBI; Maslach et al., 1996). It will be adapted for use in student samples, following Schaufeli et al. (2002). From this questionnaire we will use the scale Exhaustion (5 items). Items are scored on a 7-point frequency rating scale (0 - "never", 6 - "every day"). High scores are indicative for high levels of fatigue. We will use cut off scores that have been developed by Schaufeli and van Dierendonck (2000). Measured at T1 to T10.

-Need for recovery will be assessed with the 6-item 'need for recovery scale' (Van Veldhoven et al., 2002). Measured at T1 to T10.

-Fatigue will be measured by using the 10-item Fatigue Assessment Scale (FAS) developed and

validated by Michielsen et al. (2003). Measured at T1 to T10.

Health and well-being

-Health & Well-being: Following De Bloom et al. (2010) we will employ seven single-item measures to tap several main indicators of health and well-being: health status, mood, stress, fatigue, tension, energy level and satisfaction. Participants will report with a report mark between 1 and 10.

-Sleep quality: five-items sleep quality scale (Van Veldhoven et al., 2002). (e.g. 'Last night I woke up several times'; 1 = 'yes', 0 = 'no'). Measured at T1 to T10.

Cognitive functioning

-Executive functioning (working memory, inhibition, task switching) will be assessed by using the following measures: 2-Back task (Kirchner, 1958), the Sustained Attention to Response Test (SART) (Robertson et al., 1997) and the Matching task (Poljac et al., 2010). These are three well-validated tests, each tapping specifically into one of the three target functions (Oosterholt et al., in press). These tests will be assessed two times: at T1 and T7 (because of a possible learning effect).

-CFQ (Broadbent et al., 1982: Cognitive Failures Questionnaire). A Dutch translation (25 items) will be used to assess the participants' self-reported cognitive functioning in daily life. Measured at T1 and T7.

Self-efficacy

-Self-efficacy will be measured by the Dutch Self-Efficacy Scale (10 items, Schwarzer S Jerusalem,

1995) and one item report mark grade (on a scale from 1-10) provided by the participants (see Van Hooff et al., 2007). Measured at T1 to T10.

Participation in daily life

-We will also monitor participation in daily life (social interaction with family, friends, students, student networks). Measured at T1 to T10.

Control variables

-Exercise other than during the running of the exercise intervention. We will measure exercise activities as well as associated exercise experiences (pleasure and effort). Measured at T1 to T10.

**Did you submit a similar research to the Ethics Committee before?**

(x) No

( ) Yes, number:

**Ethics**

**1. Please find below the rules a researcher should follow according to the Ethics Committee. Following these rules is the responsibility of the researcher and the supervisor. Please indicate if you follow these rules:**

A. The study will provide new and important insights.

B. The study has the appropriate methodology.

C. The study is under supervision of an expert. Those who deliver the intervention are experts.

D. The study is not conducted among participants who are subordinates outside the study (e.g. own children).

(x) Yes

( ) No

**2. Is there someone participants can approach when there are questions about the study, and do participants know who this person is?**

(x) Yes

( ) No

**3. Is there someone participants can approach when there are complaints about the study and is it clear to participants how complaints will be handled?**

(x) Yes

( ) No

**4. Is participation entirely voluntary and may participants withdraw consent at any time without giving a reason?**

(x) Yes

( ) No

**5. Are participants informed about the goal, type, duration, risks and drawbacks of the study, and do participants sign informed consent?**

(x) Yes

( ) No

**6. If there is deception in the study, does the study meet the requirements for deception?**

(x) There is no deception

( ) There is deception, and the study does not meet the requirements

( ) There is deception, and the study meets the requirements

**7. Does the study meet requirements of anonymity and privacy?**

The data:

a. will be anonymously handled and confidentially stored

b. will be made available to participants, when they would like to have insight in their own data

c. will only be accessible for researchers that are part of the research project

(x) Yes

( ) No

**IV. Participants**

**8. Does the study concern healthy participants?**

(x) Yes

( ) No

*The participants are physically ‘healthy’. They experience (non-clinical) fatigue problems.*

**9. Are participants medically screened to diminish the potential risks of the study?**

( ) Yes

( x ) No

**10a. Is the study done among minors or people who are legally incapable?**

( ) Yes

( x ) No, please continue to question 11

**10b. Does the study concern a specific target audience?**

( ) Yes

( ) No, please continue to question 11

**10c. Is there a practical reason why minors or people who are legally incapable participate in the current study (e.g., because there are not sufficient legally capable people available to participate)?**

( ) No

( ) Yes

**10d. Is it possible to gather the required knowledge among people who are legally capable?**

( ) No

( ) Yes

**10e. Is the current study an intervention study?**

( ) No

( ) Yes

**V. Employed method**

**11. In the current study, is there a method used that could reveal new information concerning participants’ health?**

( x ) No

( ) Yes

**12. Are there unobtrusive methods used?**

( x ) No

( ) Yes

**13. Are participants subject to treatments/interventions that are beyond someone’s ‘normal lifestyle’?**

( x ) No

( ) Yes

*Participants of the current study will be asked to engage in running sessions three times a week (twice a week in a group, under supervision of a trainer; once individually). Before participating in the current research, participants do not engage in ≥ one hour of exercise a week. Participation in the intervention is voluntary. Participants are free to continue regular exercise after the intervention.*

**14. Are the risks minimal? Is it a risk participants would also run in daily life?**

(x) Yes

( ) No

**15. Is there a reimbursement given to participants that is different from the standard reimbursement?**

( x ) No

( ) Yes

**VI. Supplementary information**

Use the space below to address issues that also need to be discussed.

**VII. Supplementary forms**

Together with this form, you will need to hand in:

- Advertisements of the study
- Text of the debriefing, together with contact information of the researchers
- Copy of informed consent
- If the study is conducted externally (e.g., school, hospital), please attach an consent form signed by both the external party and the researcher.
